# Communicating climate change induced migration: the role of NGOs

**DOI:** 10.12688/openreseurope.16232.2

**Published:** 2024-03-13

**Authors:** Maria Sakellari

**Affiliations:** 1 School of Art & Media, University of Brighton, Brighton, England, UK

**Keywords:** climate change communication, migration, non-governmental organisation, discourse analysis

## Abstract

This study addresses the underexplored issue of climate migration in non-governmental organisations (NGOs) communication, which is particularly relevant given the anticipated effects of climate change on migratory patterns. It paints a richer picture of NGOs’ visual and textual discourses on climate migration and delves into the ways in which NGOs' depictions of climate migrants reinforce the 'us' and 'them' dichotomy that characterises policy and media circuits' wider debate on this issue. NGOs visual practises and textual narratives depoliticize climate migrants by underlining their otherness and propensity to bring social instability and disturbance. This raises doubts about the efficacy of climate migration-related online public education and policy advocacy efforts run by NGOs. This paper innovates as it encourages NGOs to create new ways of depicting climate refugees. It provides a framework for thinking about the role that NGOs could play in creating new ways of discussing climate migration.

## Introduction

Migration patterns are affected by climate change ( IPCC, 2021). The intersection of climate change and migration, as well as climate injustice, have been frequently cited with reference to not only the Pacific Island nations ( Dreher & Voyer, 2015), but also African ( Mastrorillo *et al.*, 2016) and South Asian ( Islam Rezaul & Shamsuddoha, 2017) countries. This is because poor populations, who contribute least to climate change, are those most dramatically and immediately affected.

Actions by international policy and authorities to address the issue remains poor, however, in a global setting where migration and refugee policies in general have become increasingly restrictive ( Greenhill, 2016). In the Paris Agreement ( UNFCCC, 2016), for instance, the term ‘displacement’ is used without being defined, and the UN Global Compact of Migration (2018) mentions climate change induced migration without guaranteeing any special protection for those who experience it. 

Key opinion shapers on the issue of climate migration include the media, government, and international bodies in addition to non-governmental organisations (NGOs). In both the highest levels of politics and media coverage of the issue, affected individuals and groups are portrayed as a danger to social cohesiveness in host countries ( Sakellari, 2021). In a global setting with minimal policy action on the issue, it is mostly NGOs who campaign for reforms connected to policy and public awareness of climate migration ( Ransan-Cooper *et al.*, 2015). NGOs have played a significant role in international climate negotiations ( Kuyper & Bäckstrand, 2016). Scholarly studies have identified cases where NGOs’ lobbying activity influenced the formation of international and domestic climate change policy in western democracies ( Allan & Hadden, 2017; Dolšak, 2013).

Those NGOs working on the climate change and migration nexus have webpages where they discuss the relationship between climate change, global inequality, and migration. Images on these sites and in these blog posts help readers grasp the scope of climate change's impact on migration. Target audiences may have never personally observed human migration amid changing climate, making visual tales even more significant. Thus, as images have been documented of being amongst NGOs’ tools to generate political discussion about action and justice ( Manzo, 2010), these visual narratives of climate migration of NGOs are intrinsically political.

Therefore, we must pay attention to the images that NGOs chose to communicate and involve the public in the larger discourse of climate change and migration, since visuals play a vital part in developing ways of knowing climate migration. Meanings of the visuals themselves is the initial point of inquiry. What do pictures on NGOs' websites and blogs reveal? The second inquiry concerns the relationship between text and visuals: How can visuals and words work together to shape migration due to climate change as a topic of discussion? In what ways does this mediation ultimately hurt underprivileged and local populations?

This research uses the example of the UK to answer these issues by analyzing the NGOs' climate migration-related websites and blog posts. Particularly in the UK, there has been a dramatic shift in attitude towards who is responsible for safeguarding the rights of climate migrants. To de-securitize climate-driven migration, the UK government funded the Foresight Project on Global Environmental Migration in 2009 ( UK Government Office for Science, 2011). This project highlighted climate migration as an adaptation response, in which people relocate voluntarily to lessen the vulnerability of communities in climate hot-spot areas. However, UK climate policies did not advance the idea of migration as an adaptive approach. Instead, migration is listed as a negative health and wellbeing impact of climate change in the first UK Climate Change Risk Assessment Evidence Report in 2012 ( UK Government DEFRA, 2012), which was produced in response to the 2008 Climate Change Act. It is only in Chapter 7: International elements of the later UK Climate Change Risk Assessment Report ( UK Government Committee on Climate Change, 2016) that migration due to climate change is mentioned at all. The UK is a highly sought-after destination for refugees and immigrants, but public discourses on migration have grown increasingly divisive in the wake of the Brexit vote ( Meleady *et al.*, 2017).

Using the UK as a case study, this research conducts a critical visual analysis of NGOs' websites and blog posts devoted to climate migration, paying special attention to the relationships between the images and the surrounding text. Since there is a dearth of academic research on climate migration in NGOs' communications, this study makes a significant contribution and fills a gap in the field. Farbotko (2005); Farbotko (2012); Herrmann (2017); Mahony (2016); Methmann (2014); Randall (2017); Sakellari (2022) are only a few of the authors that have examined policy and media portrayals. In the larger discussion about climate change and migration, NGOs have been understudied as a unique collection of actors with their own characteristics, potential, constraints, and operational rules. Findings are expected to be generalizable to other similar NGOs in other traditional migrant-receiving nations in the West because of the widespread agreement on the importance of public education, policy advocacy campaigns, and climate migration. Consequently, this emphasis on the UK provides a framework for comprehending how the visibility or invisibility of certain aspects of the issue of climate migration in NGOs' campaigns relate to questions of the political agency of communities affected, the consequences on their inherent rights, and the implications of public understanding.

## NGO’s discourses of climate change induced migration

People who are impacted by climate change and migration have been portrayed in the literature as either helpless victims of the devastating effects of climate change or as pathological sources of negative acts and criminal behaviour in host societies ( Dreher & Voyer, 2015; Farbotko, 2005; Farbotko, 2012; Herrmann, 2017; Mahony, 2016; Methmann, 2014; Randall, 2017; Ransan-Cooper *et al.*, 2015; Sakellari, 2021). Although they may appear to be diametrically opposed, victim framing and threat framing are, in fact, interchangeable. Scholarly research on the representations of climate migration in the media and policy circuits explicitly demonstrate how the figure of the climate victim is constructed as a distinct ‘Other’, which not only denies political agency, but also portrays their condition of statelessness as a security threat to the host communities ( Mahony, 2016; Manzo, 2010; Methmann, 2014; Sakellari, 2021).

Visual storytelling is acknowledged in the literature as a tool to aid in the formation of these impressions. Specifically, Herrmann (2017) conducted a critical visual analysis of US news sources that portrayed internal relocation as a result of climate change. He illustrated how pictures victimise and disempower vulnerable groups through his findings. In a comparable spirit, Methmann (2014) demonstrates how climate migrants are portrayed as racialized and passive victims of a changing climate by conducting a visual discourse analysis of pictures taken from notable reports on climate migration published by large NGOs, international policy and scientific bodies, global websites, and prominent newspapers in the UK, Germany, and the USA. The pictures were taken from reports on climate migration published by these organisations. Sakellari (2021) demonstrates how the figures of climate migrants are depoliticized in the visual representations of climate migration within the online news media in the UK. As a result of this, the figure resembles the referent objects of securitizing claims. Manzo (2010) investigates the iconography of climate change in contemporary climate action campaigns in the UK and underscores that climate migrants are typically depicted as powerless victims of a changing environment, which poses a potential risk to the community that they are living in. Mahony (2016) focuses on modern photomontage images of climate migration and highlights the fact that climate migrants "trade on equally imperial themes of lost or threatened order" (p.15).

As was discussed earlier, researchers looking into the prevalent climate migration discourses have primarily concentrated their attention on policy and media depictions. NGOs have been included as part of the sample in several different research projects on climate migration discourses ( Methmann, 2014; Ransan-Cooper *et al.*, 2015) but there is not something specific focusing explicitly on NGOs. Still, the findings indicate that a multitude of reports issued by NGOs have contributed to the construction of people who have been affected as suffering and disturbed communities that are on the move. NGOs, academics, think tanks and politicians have all communicated climate migration as a future problem, using the imagery of vast and dangerous floods of climate change induced migrants from the Global South ( Bettini, 2013; Bettini, 2014). This communication of climate migration as a future crisis has also been done by policymakers. Overall, the fact the discourses discussed in the literature are not specific to NGOs, is a gap that this paper aims to address and, thus, contribute beyond what is already known.

At this point, it is important to note that migration has also been proposed as a climate adaptation measure in academic and policy contexts. The idea behind this measure is that those who are negatively impacted would move to more prosperous areas in order to find work, provide for their families, and build up their resilience. However, within the context of adaptive migration, people who are adversely affected are categorised as economic migrants. As a result of this, they run the danger of becoming vulnerable to being exploited and discriminated against ( Bettini, 2014). NGOs have linked the adaptation discourse to their already established communication routines by endorsing planned migration in climate hotspot regions as a measure to help prevent future conflicts ( Boas & Rothe, 2016). This is because the framing of migration as adaptive did not replace NGOs' storylines of climate security. Rather, it was intended to supplement the narratives of climate security.

NGOs should create an inclusive environment that welcomes a variety of climate justice perspectives and debunk fallacious beliefs about climate change's social effects, such as migration, which are often bolstered by anti-foreigner attitudes and discriminatory practices. High polluter responsibility, vulnerable group support, protection from harm, cultural influence, and ecological functioning are climate justice ( Schlosberg & Collins, 2014). Thus, NGOs should democratise climate change discourse. This could help build and spread theoretical and practical approaches to better protect damaged individuals and communities at all governance levels.

In summary, despite evidence that NGOs have a role in the governance of climate change ( Hall & Taplin, 2007; Laestadius *et al.*, 2014; Star, 2012; Szarka, 2014), insufficient attention has been paid to how and why these conceptualizations of climate migration have been spread by NGOs. In this regard and within the context of the growing use of digital technology in the public sphere, the following section of the paper zooms in on how NGOs in the UK represent the climate change and migration nexus in their online campaigns. This representation is evaluated considering how the larger social and political context is involved in the communication and policy advocacy of NGOs. As a result, it offers a more nuanced understanding of how NGOs express and depict climate migration in their many forms of communication. In addition to this, it provides a basis for the discussion of the various tactics that NGOs could engage in to participate in the development of alternative ways of representing climate migration.

## Methods

The study examines the visuals used by the leading environmental and humanitarian NGOs in the UK to convey the topic of climate-change driven human mobility on their respective websites and in blog posts. This study relied on the structural-operational definition of NGOs established by Salamon and Anheier (1992) to set the foundational criteria for inclusion of the NGOs. According to Salmon and Anheier (1992), these organisations must share the following fundamental qualities: institutionally organised in terms of their organisational form or system of operation, separate from government, non-profit-distributing, self-governing and involving meaningful voluntary participation in operations or management. An additional major criterion for inclusion in the survey was the presence of climate migration-related content on an NGO's website. By using such wide criteria, we were able to incorporate NGOs engaged in various related fields. In particular, the survey encompassed UK-based NGOs that address humanitarian and environmental issues. Not only were environmental NGOs included in the survey, but so were many whose stated mission was humanitarian rather than environmental. This was due to the fact that many of these organisations' programmes focused on combating climate change, which in turn addressed migration, poverty, and migration.As such, the analysis included images that accompanied blog posts on climate migration by larger environmental and humanitarian NGOs with multiple international chapters that occasionally posted articles and reports to raise awareness on the connection between climate change, migration, social cohesion, and poverty, and thereby motivate readers to support their overall work primarily through attending their events, volunteering, and donating. These NGOs include Climate Outreach, Save the Children UK, UNICEF UK, Oxfam UK, and Friends of the Earth UK.

Further, the research focused on the visual content that accompanied the websites of two NGOs: the Climate and Migration Coalition and the Environmental Justice Foundation. The Climate and Migration Coalition has the first UK-based NGO website devoted exclusively to climate migration. The group promotes awareness and knowledge by use of online media like videos, webinars, and podcasts. The Environmental Justice Foundation, on the other hand, is the UK's sole environmental justice-focused non-governmental organisation and maintains a policy advocacy campaign to safeguard climate refugees. It is important to note that the Environmental Justice Foundation is a part of the Climate and Migration Coalition, therefore the two organisations are connected. Both organisations have goals and scopes that are comparable but not identical, and they each maintain their own distinct online presence. The primary call to action for Environmental Justice Foundation is for readers to sign petitions and make donations to help spread the word about the issue. The Climate and Migration Coalition encourages its audience to donate to the cause in addition to attending webinars.

Overall, the research yielded two websites, seven blog entries with a primary focus on climate migration, and a database of 60 photos. Building on Sheufle and Tewksbury's (2007) insight that perception influences issue consideration, the study employs Entman's (1993) framing theory, emphasizing the selective portrayal of reality in messages.The analytical framework for visual discourse analysis includes analysis of visual elements with individual significance (objects, settings, poses, colours), representations of social actors (proximity, viewing angle, representation as an individual or collective), and the overall visual composition. The textual framing of the photographs is then examined to see how the image relates with text to create climate migration as a discursive object, borrowing further from the critical discourse analysis methodology ( Van Dijk, 2009; Wodak & Meyer, 2015). Literature suggests the term "multimodal" to denote the incorporation of multiple sensory inputs or outputs, thus the survey also draws on Kress and Van Leeuwen's ( 1996, 2001) framework for critical analysis of multimodal discours to examine the interplay of both visual and verbal modes simultaneously. Pauwels's (2012) multimodal paradigm for website analysis is also consulted to unveil latent agendas within the websites. At this point it is worth noting that the study prioritizes generalizing findings across a broader context rather than delving into specific differences between NGOs. This approach is taken to provide insights into overarching trends within the studied period. Last, but not least, even though this a multimodal analysis, the paper starts by examining each mode independently before discussing their interplay. This allows to delve deeper into how each modality constructs meaning and influences audience perceptions ( Sheufle & Tewksbury, 2007).

The materials studied were published from December 2015 to February 2019. This time frame was chosen because it encompassed several noteworthy events relating to climate change and migration, including the Paris agreement and the Brexit referendum. More particularly, the post-Brexit period was marked by significant political and social shifts, influencing public discourse and potentially shaping the strategies employed by NGOs in communicating climate-related issues. Additionally, this timeframe aligns with global discussions around climate change, emphasizing the relevance and timeliness of the materials under examination. The convergence of these factors underscores the importance of understanding the visual and textual framing strategies employed during this specific period to comprehend the nuanced dynamics of climate change communication.

### NGO’s imagery of climate migration

Four visual tropes were identified after a critical visual analysis of the photographs. They highlight certain visual aspects of human mobility that have been used as stand-ins for the broader theme of migration in response to climate change. The four main themes consist of: (i) fragile beings (which is identical to the “victims” trope discussed earlier in the paper), (ii) destitution, (iii) iconic settings and (iv) security as a humanitarian discourse. More particularly, 32 photos were found to fall into the first visual trope, 14 images into the second, 11 images into the third, and three images into the final visual trope. More than 70% of the collected photos were taken in the Pacific Island countries, Africa, and South Asia, all of which are regions frequently mentioned as being particularly at risk from the effects of climate change.

### Fragile beings/Victims

This theme refers to the images of locals, villagers, and inhabitants, especially vulnerable communities in climate hot spot areas in the Global South, predominating across all NGOs’ online presences, whether they be dedicated sites or blog posts. Some of the depicted individuals are standing around while they wait. Other pictures depict people who are in transit, both individually and collectively, aboard boats and in other unfamiliar settings. Some in the images have already waded waist-deep into the sea to escape the rising waters. There are also photos of children who are either happy or passively waiting as adults work in parched fields or travel through regions they will soon have to leave. All these images can make viewers feel moved. Peoples’ predicaments are brought into focus by highlighting their actual experiences with the catastrophic results of extreme weather or rising sea levels. Even though many of the world’s population lives in cities on the coasts and the poor are disproportionately affected by climate change, these individuals photographed are concentrated in rural areas. Compared to the less urbanised rural and agricultural past, the city is often seen as the symbol of modernity in the West ( Mahony, 2016). This visual motif is typical of charity advertisements and perpetuates a stereotype of the Global South as a location where people live in a preindustrial era ( Methmann, 2014). 

Colonialist depictions of “naturalness”, “nativeness”, and “primitive” lives typified by a lack of modern civilization ( Methmann, 2014) are reflected in more than 80% of the images picturing climate migrants, who are typically shown in their supposedly natural habitats. The ‘colonial gaze’ of those pictures is “recycling Western visions of a temperate world that exercises dominion over tropical realms” ( Manzo, 2010, p.1003). The focus on children, for instance, is often taken to imply helplessness, vulnerability, and the need for safeguards ( Manzo, 2008). Despite its emotional impact, this visual stereotype obscures the critical role that climate migrants play in working with national and international authorities to secure their rights and ensure their freedom of movement. These opacities dehumanise societies by reducing them to a single stereotype shared by the passive, the waiting, and the helpless ( Herrmann, 2017; Methmann, 2014).

### Destitution

Images of people on the brink of rising seas or areas ravaged by extreme weather are shown. Because of their visual nature (unlike greenhouse gases), images of climate change typically bear witness to weather extremes, “but also because they can live long in social memory” ( Brönnimann, 2002, p. 89). In contrast to the western ideas of logical planning and architectural formalism, slums have become the stereotypical picture of a non-Western metropolis ( Scott, 1998). Similarly, some pictures show dangerous, non-Western cityscapes that are obviously impoverished. This visual trope conveys deterministic linkages between climate change and society through the concept of vulnerability, suggesting that climate migrants need aid but also suggesting that they have no option except to relocate. There is little evidence of intentionality in anticipating or adapting to climate change ( Methmann, 2014). The long history of colonial policies, social and economic inequalities that first put these populations in susceptible geographies stays hidden from view ( Herrmann, 2017) while the spectators see the aftermath of a natural reclaiming of a human settlement.

### Iconic settings

Iconic images of at-risk landscapes can be found in visual tales of climate migration, with tropical islands in the Pacific making up most of these locations. Despite the differences in perspective, there are recurrent visual themes between the pictures. The islands have no discernible elevations and are surrounded by water; it is simple to picture them becoming submerged as sea levels rise. The framework of landscapes, introduced by Greider and Garkovich (1994), underscores that “what is important in any consideration of environmental change is the meaning of the change for those cultural groups that have incorporated that aspect of the physical environment into their definition of themselves” (p.21). As there is no human presence in these images of flat, gorgeous, but disappearing islands, the viewers are removed from the inhabitants, the uniqueness of each community, and the geopolitical environments of the local regions. ( Herrmann, 2017). These images of climate migration hotspots, on the other hand, play into romanticised concepts of a (mostly island) paradise on the verge of extinction ( Farbotko, 2005). This shifts the blame for climate change away from those who have historically contributed the most and onto those who have historically contributed the least, as the western countries with the highest greenhouse gas emissions are also the wealthiest and least vulnerable.

### Security as a humanitarian discourse

The Climate and Migration Coalition uses this graphic motif three times on their website, including once as its primary of its website home page image. Rhetorically, a picture of a soldier in a helicopter hovering over a flooded city begs the government to intervene directly for human security. It is very similar to the other two photographs, which show soldiers surrounding civilians and a police officer in front of a gathering of refugees. Securitization, like that of society and state security ( Watson, 2011), occurs when humanitarianism is used as an excuse for the use of security and military action to obtain control. Climate change is portrayed as a dangerous hazard, a threat multiplier, which means that vulnerable, underdeveloped, and non-resilient communities must be managed, and extreme measures become necessary for their protection, in the view of Bettini (2014).

## Messages and storytelling

This section delves into how the four visual tropes are contextualised in the text. When discussing their dedication to those most in need, NGOs frequently use language centred on justice and solidarity:

“Climate change is threatening the lives of the world’s poorest and most vulnerable – displacing millions of people.” ( Friends of the Earth UK, 20 June 2017)

“While no country is safe from climate impacts, it’s the poorest and most vulnerable communities – those who did the least to cause the climate crisis – who are hardest hit.” ( Environmental Justice Foundation)

But, accompanying narratives tend to mirror the dominant discourses of climate change, that is determinism, crisis and catastrophe, where at the epicentre stands the figure of the vulnerable being ( Manzo, 2010).

“Climate change is an existential threat to humanity.” ( Environmental Justice Foundation)

“Even the most ambitious plans to halt climate change still commit the planet to more warming. That warming will continue to alter patterns of drought, flooding and sea level rise. Those disasters will displace more people in the future, regardless of how fast we cut emissions.” ( Randall 2018, Climate Outreach)

“2,500 others have also fled this group of coral atolls known as the Carteret Islands. It’s part of a growing humanitarian crisis – one that’s only set to get worse.” ( Friends of the Earth UK, 20 June 2017)

This collective threat and world problematic conceptualization of climate change and, in turn, climate driven migration is intended to motivate action; however, it erases the complexity and diversity underlying human mobility ( Manzo, 2010). In addition, migrants are portrayed as helpless and in a dire situation, as rising sea levels and extreme weather conditions compel them to migrate and those who cross borders are not eligible for asylum.

“Rising sea levels are forcing Pacific islanders to leave not only their homes but their culture, traditions and spirituality embedded in their land.” ( Kennerley, Friends of the Earth UK, 21 June 2018)

“Trump’s threat of a border wall, and an increasingly hostile environment for migrants in the US makes journeys like this harder and more dangerous.” ( Randall, Climate and Migration Coalition).

However, this emotional portrayal omits the historical and political context of migration, such as the lengthy history of colonialism in vulnerable areas. It obscures the fact that many people’s vulnerability to climate change is a result of their everyday existence, and instead presents climate migration unrelated to social systems.

Alternatively, a campaign may appear to present contradictory interpretations of climate migration. For instance, on the Climate and Migration Coalition website when speaking about climate migration in Latin America, it is acknowledged that:

“Whilst there are inevitably a range of factors that lead people to migrate, the impact of climate change, especially if livelihoods are damaged, may intensify rural-urban migration.” ( Randall, Climate and Migration Coalition)

However, in a story about climate migration in the Sahel region, a long history of colonialism and western interventions that exacerbated socio-economic vulnerabilities to climate change remains neglected:

“The Sahel region is highly dependent on agriculture for livelihoods and the wider economy.... Over the past half century a combination of land degradation, population growth and misplaced environmental and development policies have contributed to vulnerability.... So while it is impossible to state that climate change caused a particular drought, it also not the case that climate change has no effect.” ( Climate and Migration Coalition)

Voices of people from vulnerable areas are given authority in demonstrating the effects of climate change.

“Last year Chief Dearling led his community in the traditional rain dance ceremony but he recalls “no rains came, we lost all our cattle and we could not plant anymore. This means I have failed my community.” ( Kennerley, Friends of the Earth UK, 21 June 2018)

Nevertheless, this conception of climate migrants as victims obscures their agency. Literature on NGOs' campaigns demonstrates that NGOs' advocacy for issues that elicit strong visceral responses from their target audiences disempowers local communities ( Cameron & Haanstra, 2008; Chouliaraki, 2013; Fernandez-Aballi, 2016). NGOs represent migrating people as relying on the help of environmental charities to reach their political goals:

“EJF is working tirelessly to give these vulnerable communities a voice and secure international protection for climate refugees.” ( Environmental Justice Foundation)

“We exist to protect the rights of anyone facing these circumstances.” ( Climate and Migration Coalition).

This, however, has been identified as a typical pitfall among white social movements ( Salter, 2013). When advocacy campaigns rely on the logic of northern-based NGOs as protectors and saviours of people and communities from the global South, their campaigns become carriers of imaginaries that perpetuate neo-colonial discourses of Western hegemony ( Cameron & Haanstra, 2008; Chouliaraki, 2013).

Accompanying narratives imply that climate change can have significant implications for social stability by potentially increasing the likelihood of social unrest and conflict.

“Despite the huge sense of urgency these situations bring, and the dangerous links between climate change, insecurity and migration and refugee flows, not enough is being done to coordinate international processes on climate change and migration.” ( Alfaiate, Save the Children UK, 14 December, 2017)

“Drought and extreme storms are forcing people to leave their homes in search of a better life. And there is an increasing risk of communities clashing over precious natural resources such as water.” ( Friends of the Earth UK, 20 June 2017)

Nonetheless, there is limited and, at times, exaggerated evidence that climate change will cause conflicts ( Frohlich, 2016; Selby *et al.*, 2017). This is consistent with the colonial perception of the South as a place of paucity and disarray ( Selby, 2018). In order to address climate-related migration, NGOs use a threat-urgency approach. The prevalent narrative depicts displaced individuals as being in danger from a host of issues, such as a lack of shelter, riots and violence, and damaged infrastructure. That is why this narrative sounds like a story about securing something. Specifically, the humanitarian crisis homogenises people into helpless recipients and victims. As such, we ‘should’ resort to extreme measures in order to 'defend' them if their native country is unable to do so. The humanitarian justifications used by the USA and UK elites for the war of Iraq ( Watson, 2011) are an example of this humanitarian securitization logic.

A disjuncture between image and content exists on the Climate and Migration Coalition website. Rather than being complementary, certain images do not directly correspond to the content of the website. On the website of Climate and Migration Coalition, it is acknowledged that:

“the relationship between climate change and armed conflict is complex. Some evidence points towards changes in weather patterns leading to upticks in armed violence and conflict’’ …. the real risk is to migrants and refugees themselves, rather than migrants creating security risks in the places they move to”

The website's homepage and other photographs in the picture gallery, however, depict security or military activity performed in response to calamities or population displacement. Images like these, which use the language and tactics of security to portray climate migration, give viewers context for understanding the stories that go along with them, and may end up clouding rather than clarifying policy proposals meant to mitigate the social effects of climate change. There is debate over whether the best way to deal with climate migration is to help displaced people find new jobs, relocate, or have the government step in.

In general, for the NGOs examined here, one of the ways of assisting countries in the Global South to cope with climate change effects on the human population is through international legal agreements that facilitate mobility and planned relocation actions.

“Right now, migration is one of the most important homegrown climate adaptation strategies. European leaders now have to work out how to facilitate this. It will not be climate change that creates another refugee crisis. Rather, it will be attempts to stop this migration that creates a crisis." ( Randall, Climate Outreach)

“EJF calls on governments to recognise climate refugees and support a new legal agreement to guarantee their rights and their fair claim to our shared world.” ( Environmental Justice Foundation)

People of different backgrounds, ethnicities, socioeconomic standings, professional experiences, and future goals are often grouped together when discussing migration as a means of adaptation. Although these adaptation narratives exist alongside recognition of the rights and interests of vulnerable communities, they nonetheless assume their reliance and call attention to their otherness. It also allows us to critically reflect on the kind of humanitarianism embedded in NGOs discourses, in which:

“certain actors are privileged in the identification of both human insecurity and the appropriate measures for ‘restoring’ affected humans to a condition of security” ( Watson, 2011, p.15).

Overall, the political aspect of NGO messages, which demand action from both politicians and ordinary citizens, is reflected in the use of images of human vulnerability in NGO advertising. Accompanying words, however, fail to capture climate migrants' political agency. Little is understood, for instance, about the measures vulnerable groups take to save livelihoods, defend homeland and culture, and especially the decision to stay, to deliberately remain stationary ( Farbotko, 2018). NGOs' visual narrative of victimhood speaks to a personalised and emotionalized perspective of climate-driven human migration, but the writings that accompany the images don't address the reasons why some people and some nations are more vulnerable to the effects of climate change.

## Discussion

According to the data, NGOs do use a unified, central image when discussing climate migration. Findings from other studies on NGOs' visual discourses of the social aspect of climate change ( Manzo, 2010; Methmann, 2014) are consistent with the observation that images of people migrating due to climate change are disproportionately concentrated in the poor countries of the Global South. A long history of neglect and colonialism by the wealthier countries of the Global North has exacerbated socio-economic vulnerabilities to climate change ( Farbotko, 2005; Herrmann, 2017), but the North is portrayed as a haven, capable of throwing a lifeline to the struggling South. As a result, policy visions to address and fix the fundamental causes of vulnerability within at-risk communities are left out, and the colonial policies that initially positioned climatic hot-spot places in susceptible geographies remain hidden. In addition, the Western audience's perception of NGOs is reinforced because of the emphasis they place on disadvantaged people and communities in developing countries. It is important to point out the parallel with a problematic aspect of climate photography in general: the disproportionate portrayal of regular people as climate change victims ( Wang *et al.*, 2018).

The political agency of climate migrants is not fully represented within NGOs' accompanying narratives, even though they promote the notion that climate change is a justice issue and, hence, political ( Manzo, 2010). The inclusion of climate migrants' voices in NGOs' written narratives about climate migration is meant to provide more positive, sympathetic, or humanising depictions of migration; however, these voices portray the migrant as a victim in need of support and sympathy rather than a person who can challenge political or expert authorities. Those who are affected are collectively viewed as a group of victims who are all the same regardless of their origins, beliefs, or socioeconomic standing. While NGOs let their audiences imagine that climate change induced human mobility is driven by something very visible, a climate-driven disaster like a hurricane, flood, or drought, much of it is driven by less obvious forces like land degradation and biodiversity loss, which are often overlooked in NGOs' narratives about climate migration. Human migration due to land salinization or species extinction is not as sudden, widespread, or tragic as that caused by extreme weather. It is important to note at this juncture that some experts in the field of climate change communication worry that the catastrophic framing of this issue sends the wrong message to the public ( O’Neill & Nicholson-Cole, 2009; Sakellari, 2015). Overarchingly, NGOs strategically use the plight of vulnerable populations to fight for their interests and to generate donations. These communities are viewed as a distinct and distant 'Other' and their plight is presented as such. The neoliberal logic of pity, guilt, or authenticity are used in advocacy campaigns as a means of increasing brand legitimacy and funding, but they do not get at the root causes of the defined problem ( Fernandez-Aballi, 2016).

NGOs' portrayal of climate refugees is consistent with that of the media, government policy, and other sectors. Many who advocate for climate refugees tend to view the 'Other' as a victim as well as a potential threat ( Baldwin, 2014). This could lead to policy developments that aren't relevant to those who need to relocate, or even risk increasing xenophobia and authoritarian policies, as pointed out in the political economy ( Bettini, 2013; Bettini, 2014), development studies ( Hartmann, 2010), and media studies ( Sakellari, 2021; Sakellari, 2022). As Miller (2017) underscores a sustainable national security policy tends to prevail as a climate adaptation policy for the rich and powerful.

NGOs can use adjustments in discourse as a powerful weapon to affect governance results. Allan and Hadden (2017), for instance, highlight how NGOs have increased their influence at the UN Paris discussions by increasing their mobilisation and adopting a climate justice framing of climate change, with a specific emphasis on loss and harm. However, this raises the question of how NGOs might adapt their communications on climate migration to break down the 'we' *versus* 'them' binary, consider how those directly impacted by the issue frame their experiences, give voice to local actors, and ultimately bring about positive political change. Because of this, NGOs' communications should highlight the political and economic systems that render some groups less resilient and less capable of recovering from the stressors brought on by climate change and reflect the multifaceted nature of migrants' and migrants' society, including its wide range of countries, ethnicities, socioeconomic backgrounds, and other identities ( Bettini, 2013, Bettini, 2014; Sakellari, 2021; Sakellari, 2022). Furthermore, NGOs may captivate audiences by sharing a personal experience, but this narrative needs to be set in context. They need to look at the big picture and explain how political and societal factors play into the issue at hand. For instance, if there is already a lack of political stability, democratic governance, and social welfare, climate change may lead to a conflict ( Abel *et al.*, 2019). By taking this route, NGOs can connect with and potentially win over marginalised populations that otherwise feel their opinions aren't heard ( Dreher & Voyer, 2015).

The compassion and care that can stimulate introspection on the interconnected nature of vulnerabilities is worth safeguarding from the emotionalism that is inherent in both the victim and danger frames, as suggested by Ransan-Cooper *et al.* (2015). For this reason, NGOs' communication of climate migration needs to be widened to consider not only the unique challenges faced by climate migrants, but also the broader social and political issues that impact both migrants and their host communities. Those who need to relocate will be better able to integrate into their new communities if they can move past the ‘us’ and ‘them" mentality and instead focus on building relationships based on mutual respect and compassion.

Since the climate change and migration debate is extremely political ( Baldwin, 2014), the NGOs' overarching goal should be to facilitate the democratisation of this debate through their shift in discourse. To achieve this goal, NGOs' communications must take into account not only the complexities of human mobility decisions brought on by climate change, historical injustices, imposed adaptation burdens, and universal human rights, but also the right of vulnerable communities to determine whether or not adaptive migration strategies are in their best interests, and the role that local factors such as governance, tradition, culture, and access to decision-making processes play in this process ( Bettini, 2014). HaveYourSei is a real-world illustration of all the above suggestions in action. Through this initiative, Pacific islanders shared their stories of triumph and struggle in the face of climate change through photographs and spoken word. These affected communities' attempts at communication reveal a new information economy that can guide NGOs towards a more nuanced and empowering portrayal of marginalised groups ( Herrmann, 2017).

In overview, this study offers an in-depth examination of the understudied but critical role of NGOs in shaping climate migration discourses and aims to inspire NGOs to portray climate refugees differently. It suggests integrating more climate migrants' perspectives, such as a local agency that promotes community advocates as empowered leaders, to provide further interpretations. It advocates using shared identity and vulnerability interconnection to make mobility options in response to climate change. This narrative shift could help NGOs influence policy and public awareness of climate migration to defend climate migrants' rights and interests. It should be noted, however, that the study concentrates on a specific time period in terms of NGO’s communication. This poses a limitation for the findings of this study in a world of rapid and constant information flow and shifting forms of communication. Moreover, as the broader debate on climate migration remains controversial in the public sphere ( Sakellari, 2022), the study's findings are subject to additional limitations.

## Conclusion

While immigration has been a part of the environmental movement for some time, the connection between migration and environmental challenges has taken on new significance with the advent of climate change ( Farbotko 2005; Sakellari, 2022). This paper addresses a gap in the existing literature on climate migration discourses by delving into the under-researched but crucial role that NGOs representations play in establishing a public narrative of climate migration. This article contributes empirically by analysing NGOs’ communication in the UK, a long-standing haven for refugees and migrants and one of the places where the security argument of climate migration is played out in real politics. 

The fragile human being is often depicted as the focus of nongovernmental organisation depictions of climate migration. These visual practises are accompanied by textual narratives that depoliticize climate migrants by emphasising their otherness and ability to cause social chaos and disruption. Analyses of climate migration representations in policy and media circuits have found that vulnerable persons are often cast in the role of the powerless universal subject of climate migration advocacy, which is consistent with this finding. This article demonstrates that this raises doubts about the efficacy of climate migration, online public education and policy advocacy efforts run by NGOs.

This study innovates as it encourages NGOs to create new ways of depicting climate refugees. It recommends including a wider variety of climate migrants' perspectives in order to provide a wider range of possible interpretations, such as that of a local agency that portrays community advocates as empowering leaders. It suggests that while making mobility decisions in response to climate change, people should appeal to a shared sense of identity and the interconnectedness of vulnerabilities. To better protect the rights and interests of climate migrants, this change in narrative could aid NGOs' initiatives to influence policy and public understandings of migration due to climate change.

Future research could concentrate on which images gain the most resonance and traction, such as those that successfully spread beyond NGOs' reports and websites to social media feeds or mainstream media coverage of these reports. The topic of inference warrants additional investigation. In addition, there is a need for more study into the conditions that foster NGOs mediation, as well as their political economy. Scholars of environmental and climate change communication now have a chance to critically evaluate the role that NGOs played in shaping a climate change communication paradigm that benefits some campaigns at the expense of those who would be most negatively impacted by this issue. Finally, although the paper is primarily situated within a relatively small body of literature on climate migration, findings may contribute to research on multimodal analysis and social movements and be generalizable to other NGOs in the West because of the widespread agreement on the importance of public education, policy advocacy campaigns, and political agency of vulnerable social groups ( Fernandez-Aballi, 2016).

## Ethics and consent

Ethical approval and consent were not required.

## Data Availability

Data consist digital visual and textual content publicly available and retrieved from: i) the entire website of the https://climatemigration.org.uk/ ii) the Environmental Justice Foundation’ website section dedicated to climate migration: https://ejfoundation.org/what-we-do/climate/protecting-climate-refugees, as well as the hyperlinks embedded in the textual content of this particular section iii) Randall, A. (2018) “What we get wrong about migration and climate change”, blogpost on Climate Outreach’s website and available at: https://climateoutreach.org/what-we-get-wrong-migration-climate-change/ iv) Alfaiate, J. (2017) “Migration. Refugees and Climate Change: Big problems need big solutions”, opinion article posted on Save the Children’ website, available at: https://www.savethechildren.org.uk/blogs/2017/migration-refugees-climate-change-big-problems-require-big-solutions v) “Climate refugees”, article posted on Friends of the Earth’ website on 20 June 2017, available at: https://friendsoftheearth.uk/climate/climate-refugees vi) Kennerley, R. (2018), opinion article posted on Friends of the Earth’ website and available at: https://friendsoftheearth.uk/climate/climate-refugees-people-behind-climate-change-numbers vi) “Displaced by the climate crisis: voices from the field”, piece from Oxfam UK’s website, available at: https://www.oxfam.org/en/displaced-climate-crisis-voices-field vii) Randall, A. (2017) “Climate migration will only be a crisis if we make it into one” blogpost on Climate Outreach’s website and available at: https://climateoutreach.org/climate-migration-will-only-be-a-crisis-make-it-one/ ix) “No place to call home: Protecting children’s rights when climate forces them to flee”, piece from UNICEF UK’ s website, available at: https://www.unicef.org.uk/publications/no-place-to-call-home/
