# Supplementary material for: Communicating climate change induced migration: the role of NGOs
Source: Open Res Eur. 2024 Mar 13;3:163. Originally published 2023 Sep 26. [Version 2] doi: 10.12688/openreseurope.16232.2 (PMC11208855; doi:10.12688/openreseurope.16232.2)
Supplement: Peer review history

# Reviewer report: Reviewer response for version 2

**DOI:** 10.21956/openreseurope.18903.r41413

**Published:** 2024-06-26

**Authors:** Jouni Paavola, Gazi Laila

**Affiliations:** 1 School of Earth and Environment, University of Leeds, Leeds, England, UK; 2 University of Leeds Faculty of Environment, Leeds, England, UK

This manuscript examines how UK NGOs communicate climate change induced migration, drawing from visual analysis of their online materials. The manuscript clearly has potential to make an academic contribution, but it also has weaknesses which need to be addressed.

 The introduction examines the intersection between climate change, migration, and the role of NGOs in shaping public discourse and policy responses. However, it is rather dense and breaking it into smaller, more focused paragraphs would improve its readability. For instance, the discussion of the global impact of climate change could be separated from the discussion on policy responses and the role of NGOs. More clear and explicit statement of research objectives / questions and how the study advances existing scholarship would also further improve the clarity and focus of the introduction.

 The literature review section discusses research on climate migration, referencing studies focused on vulnerable populations in Pacific Island nations, Africa, and South Asia rather comprehensively. The focus on NGOs as key actors in climate migration discourse is sound. The work aims to address the gap in existing research by analysing NGOs' online communications and visual narratives in the UK, which is justified in light of the lack of earlier research in this area. However, the rationale could be strengthened by explicitly stating how the study's findings will contribute to filling this gap. Clarifying the specific research questions and objectives early on would provide a clearer roadmap for the study and help readers understand its intended contributions more effectively.

 The literature review would also benefit from a more critical engagement with the literature on NGO roles and contributions related to climate migration. While the section sets up the rationale for focusing on NGOs, it does not bridge the gap between research on media and policy portrayals and the specific contributions of NGOs in this context. A more nuanced discussion on how NGOs' characteristics, strategies, and limitations shape their communications on climate migration would better justify the study's focus and underpin it. Also, discussing recent shifts in NGO strategies or emerging critiques of mainstream narratives on climate migration would add depth to the theoretical framework. Integrating perspectives from climate justice and environmental communication research could also enrich the analysis by providing alternative lenses through which to interpret NGO communications.

 The approach outlined in methods section for conducting a critical visual analysis of NGO websites and blogs is appropriate for addressing the research questions. However, the manuscript could provide more detail on data collection methods, sample selection criteria, and analytical frameworks used for interpreting visual and textual data. It also appears as if the manuscript would move on to “the results” after the methods section but the heading structure seems to be missing a higher level heading – the subsections after the methods all appear to be about results but this is not communicated well. Some clarification here would be in order to create founded expectations about the structure of reporting.

 The discussion section is currently almost entirely geared towards communicating messages about how NGOs should / should not communicate about climate change induced migration. This obviously needs to be done in the section, but it also needs to explain how the research objectives / questions posed earlier in the manuscript have been achieved / answered, and how the manuscript advances the scholarship in the bodies of literature it contributes to. These two sets of issues should be addressed first in the discussion section, after that the practical communications ramifications currently covered can be presented.

If applicable, is the statistical analysis and its interpretation appropriate?

Not applicable

Is the study design appropriate and is the work technically sound?

Yes

Is the work clearly and accurately presented and does it engage with the current literature?

Partly

Are the conclusions drawn adequately supported by the results?

Yes

Are sufficient details of methods and analysis provided to allow replication by others?

Partly

Are all the source data and materials underlying the results available?

Yes

Reviewer Expertise:

Environmental governance related to climate change adaptation and nature conservation, environmental justice, institutional analysis

We confirm that we have read this submission and believe that we have an appropriate level of expertise to confirm that it is of an acceptable scientific standard, however we have significant reservations, as outlined above.

# Reviewer report: Reviewer response for version 2

**DOI:** 10.21956/openreseurope.18903.r38905

**Published:** 2024-05-06

**Authors:** Mark C. J. Stoddart

**Affiliations:** 1 Memorial University Newfoundland, Newfoundland, Canada

The author has made some minor revisions in response to my prior comments that go some way to addressing those concerns, and I think the article is improved as a result. But the level of engagement with and integration of my prior comments (including engagement with the suggested additional literature) have been relatively minimal. As they carry on this program of research, I would encourage the author to reflect further on these issues, including the methodological issue of the problem of inference; the interpretive issue of how climate induced migration intersects with the longer history of immigration as a source of anxiety and contestation within the environmental movement; and the interconnections between climate migration and national security discourse.

If applicable, is the statistical analysis and its interpretation appropriate?

Not applicable

Is the study design appropriate and is the work technically sound?

Yes

Is the work clearly and accurately presented and does it engage with the current literature?

Partly

Are the conclusions drawn adequately supported by the results?

Partly

Are sufficient details of methods and analysis provided to allow replication by others?

Yes

Are all the source data and materials underlying the results available?

Yes

Reviewer Expertise:

Environmental sociology; social movements and political sociology; media and communication

I confirm that I have read this submission and believe that I have an appropriate level of expertise to confirm that it is of an acceptable scientific standard.

# Reviewer report: Reviewer response for version 1

**DOI:** 10.21956/openreseurope.17522.r35387

**Published:** 2023-11-08

**Authors:** Mark C. J. Stoddart

**Affiliations:** 1 Memorial University Newfoundland, Newfoundland, Canada

Overall, this is an interesting contribution on the ways in which NGOs use visual communication to depict climate change-induced migration and immigration. I see four particular substantive issues that could be addressed to help improve the manuscript, mostly along the lines of interpretation of results and the literatures engaged with:

1. There is a problem of inference that isn't really addressed. The data collection and analysis is limited to publicly accessible NGO-produced images and texts, with little data provided on either NGO communication strategies (e.g., via interviews) encoding or audience interpretations or frame resonance. On the latter point, do you have evidence for which images (or categorical types of images) find more resonance and traction. For example, which images are most successfully recirculating beyond NGO reports & websites to social media feeds or to mass media coverage of these reports? At the least, the problem of inference should be flagged as a qualification and area for deserving further inquiry.
2. The paper is mostly situated within a relatively small body of literature on climate migration. But, more could be made about the broader theoretical contributions to research on visual analysis and social movements, going beyond this case study?
3. On the theme of securitization, the interpretation and discussion would benefit from engaging with Todd Miller's book, which goes into a great deal of depth on climate displacement as a growing rationale for hardening borders and increasing surveillance.
4. The literature review and discussion/interpretation of results would also benefit from engaging with the prior history of debates/contestation over immigration within the environmental movement. It is worth noting that this is not an entirely new phenomena related to climate change, though the re-emergence of migration in relation to environmental issues has new dimensions with climate change.

If applicable, is the statistical analysis and its interpretation appropriate?

Not applicable

Is the study design appropriate and is the work technically sound?

Yes

Is the work clearly and accurately presented and does it engage with the current literature?

Partly

Are the conclusions drawn adequately supported by the results?

Partly

Are sufficient details of methods and analysis provided to allow replication by others?

Yes

Are all the source data and materials underlying the results available?

Yes

Reviewer Expertise:

Environmental sociology; social movements and political sociology; media and communication

I confirm that I have read this submission and believe that I have an appropriate level of expertise to confirm that it is of an acceptable scientific standard, however I have significant reservations, as outlined above.

## References

[1] : Protest Movements and Spectacles of Death: From Urban Places to Video Spaces.35: 10.1108/S0163-786X(2013)0000035009 105-133 10.1108/S0163-786X(2013)0000035009

[2] : Picturing Protest. *American Behavioral Scientist* .2012;56(2) : 10.1177/0002764211419357 223-243 10.1177/0002764211419357

[3] : Toward a Visual Analysis of Social Movements, Conflict, and Political Mobilization.35: 10.1108/S0163-786X(2013)0000035004 xi-xxvi 10.1108/S0163-786X(2013)0000035004

[4] : Social and political convergence on environmental events: the roles of simplicity and visuality in the BP Oil Spill. *Can Rev Sociol* .2014;51(3) : 10.1111/cars.12045 216-38 10.1111/cars.12045 25296434

[5] : "The End of Population-Environmentalism: Dissonance Over Human Rights and Societal Goals." In Handbook of Anti-Environmentalism, edited by David Tindall, Mark C. J. Stoddart and Riley E Dunlap, 345-364. *Edward Elgar.* .2023;

[6] : Storming the Wall: Climate Change, Migration, and Homeland Security. *San Francisco: City Lights Books.* .2017;

[7] : Environmentalists' Mediawork for Jumbo Pass and the Tobeatic Wilderness, Canada: Combining Text-Centred and Activist-Centred Approaches to News Media and Social Movements. *Social Movement Studies* .2015;14(1) : 10.1080/14742837.2013.831344 75-91 10.1080/14742837.2013.831344

[8] : "Calling for confrontational action in online social media: video activism as auto-communication." In Mediation and Protest Movements, edited by Bart Cammaerts, Alice Mattoni and Patrick McCurdy, 159-178. *Bristol, UK: Intellect.* .2013;

# Reviewer report: Reviewer response for version 1

**DOI:** 10.21956/openreseurope.17522.r35903

**Published:** 2023-11-01

**Authors:** David Durand-Delacre

**Affiliations:** 1 Environment and Migration: Interactions and Choices Division (EMIC), United Nations University Institute for Environment and Human Security (UNU-EHS), Bonn, Germany

This paper analyses the text and images used by UK-based environmental and humanitarian NGOs in content pertaining to "climate migration". The author analyses two websites of NGOs highly active on this topic, and blog posts from other NGOs only tangentially active on the topic. It identifies four key themes (fragile beings, destitution, iconic locations, and security as a humanitarian discourse) in the imagery, and looks at how the images are contextualized (i.e seen against the accompanying text)

 The rationale and overall approach of the paper are sound, and the focus on NGO discourses of climate migration worthwhile as it is indeed understudied. However, I think the current version of the paper falls short of some of its stated ambitions. In particular, I am missing a genuinely joint analysis of text and image (although this is done in places). My comments are detailed below section by section. I do not find anything fundamentally objectionable in the paper (i.e. misleading or wrong) but argue that some significant modifications are required to better demonstrated the added value of the paper and improve the quality of the analysis.

**NGO'S DISCOURSES SECTION**

- The second paragraph reads as a list but it is unclear what the overarching message/takeaway should be. Simply changing the opening sentence to make this explicit could fix this.
- It is not always completely clear if the discourses discussed are specific to NGOs or observed in media/policy circles more generally. If there is something specific about NGO discourses. It might be worth making this extra explicit, so it is clear (a) what we know about NGO discourses specifically and (b) what this paper can thus contribute beyond what is already known.
- There is no discussion of climate justice discourses, which feels odd given that NGOs in the climate change space are among the most vocal proponents of such framings. Might this be missing? (I return to this later as it is part of the discussion)

Minor thing

- What does "already established routines" mean/refer to?

**METHODS SECTION**

 This section I think deserves significant reworking, as it is lacking important details.

- The structural definition of NGOs is not stated explicitly. What is it and how did it influence the sample?
- The statement that NGOs were differentiated into categories (environmental, humanitarian) is not followed up with an explicit categorisation, It would be good to know what categories each NGO has been assigned.
- The presentation of the Kress and van Leeuwen analytical framework does not explicitly explain how textual and visual analysis should be combined in their method. More details would be desirable on how this has been applied to the sample.
- Similarly, Pauwel's multimodal method is not explained at all - so it is unclear how the subsequent analysis applies it/what is learned from the application of this method?

Minor things

- By "the study was conducted between Dec. 2015 and Feb 2019", I think you mean the materials studied were published during that period? Good reasons are provided why this period is interesting, but why does it not extend further back and/or closer in time?
- The point about NGOs varying in size is unrelated to anything in the rest of the paper. Did it serve as a selection criterion? Was NGO size a factor in the analysis of the imagery and textual discourses? If yes, please elaborate somewhere in the paper. If not, why mention it?

**ANALYSIS - NGO IMAGERY**

- The breakdown of themes from the CMC website sums up to 60 pictures - which is also the number of pictures in the overall database? Something is unclear here.
- Only the CMC website is mentioned, what about the EJF one?
- Including a table or simple graph presenting each of the four themes in relation to their source (and any other significant) would aid in comprehension of the breakdown.
- Generally, the author states that images "are shown" and "can be found" but generally does not state where or whether specific NGOs employ particular themes more than others. This would be good to know and improve the quality of the analysis.
- It is not clear to me if and how the "fragile beings" trope should be considered as different from the typical/well-described "victims" trope.

**ANALYSIS - MESSAGES**

- The promise of the paper as stated in the introduction and methods is to combine the analysis of the images and text. However, the textual discourses presented in this section are mostly discussed in isolation from the images, up until the (very interesting) point about the "disjuncture" between words and images on the CMC website.

**DISCUSSION**

- Overall, the analysis does not differentiate between NGO types (even though the distinction between environmental and humanitarian NGOs was mentioned in the methods as a relevant category). Is this because all of the NGOs in the sample adopt the same tropes/themes in the same way? This needs to be made more explicit.
- As this section makes clear, NGOs do push climate justice narratives in the climate change space. Why then does this seem absent from NGOs coverage of climate migration? Is this specific to CM as a topic? Is it because we are talking about different NGOs? Any insights the author can provide on this based on their sample would be useful.

If applicable, is the statistical analysis and its interpretation appropriate?

Not applicable

Is the study design appropriate and is the work technically sound?

Yes

Is the work clearly and accurately presented and does it engage with the current literature?

Partly

Are the conclusions drawn adequately supported by the results?

Partly

Are sufficient details of methods and analysis provided to allow replication by others?

No

Are all the source data and materials underlying the results available?

Partly

Reviewer Expertise:

I am an expert on climate mobilities, specialising on issues of representation and science-policy. I have studied the climate migration discourses of French NGOs (among other stakeholders) as part of my PhD thesis (2022).

I confirm that I have read this submission and believe that I have an appropriate level of expertise to confirm that it is of an acceptable scientific standard, however I have significant reservations, as outlined above.
